# Report of Cases

**Published:** 1849-09

**Authors:** B. I. Raphael

**Affiliations:** Attending Surgeon to the Louisville Marine Hospital


Art. III—*Report of Cases.* By B. I. Raphael, M. D., Attending Surgeon to the Louisville Marine Hospital.

Case I. *Depression of Skull—Trephining.*—Geo. Goldthorp, æt. 29, boatman, born in England, was admitted in the hospital, May 23d, 1849. About nine months ago he received an injury of the head, caused by the falling of a spar. The scalp was laid open to the extent of nearly three inches, and a portion of the *left* parietal bone depressed; the fracture being about one a half inches in length, parallel with the sagittal suture, and one inch from it, half an inch in breadth, and one-fourth of an inch in depth. No attempt was made at the time of the accident to raise the depressed bone. For six weeks he had hemiplegia of the *right* side, accompanied by loss of speech. His ideas were not incoherent, but he was unable to communicate them by any possible means to another. He knew the letters of the alphabet, and could mentally connect them into words, but it was impossible for him to write words of even one syllable. It always appeared to him that there was a great weight on his head, "like," as he expressed it, "a thousand of brick." His urine was secreted in great abundance, and of a very muddy color; micturition had to be performed six or seven times during the night.

Besides these symptoms, he has had two epileptic attacks; one about three, and the other about four months after the occurrence of the accident. He has also had frequent symptoms of an approaching paroxysm. His bowels are always costive, and are only moved when acted upon by some cathartic medicine. The history of the case, so far, was given by the patient himself, and he desired that an operation might be performed. He was well aware of the danger attending it, but said he would rather risk his life than have another epileptic attack.

*Actual condition, July* 10, 1849.—Depression of the *left* parietal bone, situated one inch from the median line, and two inches beyond the coronal suture; its measurement as follows: one and a half inches in length, half an inch in breadth, and about one-fourth of an inch in depth. Pupils: the left one natural, the *right* slightly dilated, and the iris more sluggish in its movements than that of the opposite side. Pulse 94, full, soft and regular; tongue healthy; bowels constipated. Partial hemiplegia of the *right* side, with diminution of temperature.

*Operation.*—At 12, M., I performed the operation of trephining after the usual manner, in the presence of my friends, Drs. Gross, Richardson and others. The skull was half an inch in thickness, and the depressed portion had left a corresponding impression on the dura mater, to which it was also slightly attached. No hemorrhage of any consequence followed the operation. The patient expressed himself as feeling better, so soon as the depressed bone was removed. Pulse the same as before the operation. An hour after, the flaps were drawn together with adhesive strips, cold water dressing was applied, and a purgative of calomel and rhubarb administered.

11th, 10 A. M. Pulse 90, soft and regular; tongue healthy, skin moist; both pupils of the same size, and natural; some thirst; pain above the eyebrows; and a feeling of soreness in the paralyzed side. The medicine which he took yesterday had operated well.

Cold applications to the head continued.

About noon he had an epileptic convulsion, which lasted two minutes. The spasmodic action of the muscles in the paralyzed leg gave the first indication of the paroxysm. This spasmodic action soon became general and the whole body violently convulsed. Towards the close of the paroxysm his face became very livid. Pulse, half an hour after, full and soft. Patient in other respects about the same as before the paroxysm.

12th. Pulse 122, and soft; face flushed; conjunctivas a little congested, pupils natural; tongue coated with a white fur; skin moist; twitching of the muscles of the right side of the face; slight delirium. ℞ Ant. Tart. gr. j., spts. Minder. ℥iv. A tablespoonful every two hours.

4 o'clock, P. M. Pulse 130, soft and compressible; seemed to be in a state of stupor; could be aroused, but would not answer questions when spoken to; face flushed; skin moist; erythema on the superior part of the chest and anterior part of the neck. During the day has had two slight epileptic paroxysms. Bowels moved several times during the morning.

Treatment continued.

Visited him again at 8 o'clock, P. M. Condition much the same as at 4 P. M. Has had spasmodic contractions of the muscles in various parts of the body; particularly of the muscles supplied by the right facial nerve. He was bled to the extent of ℥xxx, which considerably reduced his pulse.

13th. Pulse 128, very weak and irritable; face flushed; pupils a little dilated and sluggish in their movements; skin soft and moist; would not protrude his tongue, or pay the least attention to what was said to him; insensibility of the *right* leg, but not an entire loss of motion; erythema subsiding; had two healthy evacuations during the night; slight tumefaction of the wound; all except the angle has united by the first intention. The dressings were removed, and some coagulated blood escaped, together with a little serum. The angles of the wound were opened, and a flaxseed poultice applied; the cold applications continued to the surrounding parts. ℞ Prot. Chlor. Hyd. ʒss. M. et div. in char. vj. One of these powders to be given every four hours. The spts. Minder. and ant. tart. to be continued.

7 o'clock, P. M. Condition much better; pulse 118, full, soft, and regular; patient a great deal more rational; face not so much flushed; skin moist; tongue coated in the centre with a brownish fur; pupils natural; bowels soluble, and evacuations healthy; sensation returning in right leg; would give monosyllabic answers when spoken to.

Treatment continued.

14th. Condition much improved since yesterday; pulse 94. His improved condition continued throughout the day.

Same treatment continued, with the exception of the calomel, which was omitted.

15th. Pulse 86, full, and firm; tongue coated with a white fur; skin soft and moist; pupils natural; expression of countenance better, but staring; more rational, but cannot converse; had two tolerable healthy evacuations during the night; twitching of the muscles of the *right* side of the face. ℞ Prot. Chlor. Hyd. gr. xij., Pul. Ipec. gr. iij., M. et div. in char. vj. One every four hours. Poultice to the wound and cold to the head continued.

16th. Condition much worse; pulse 100, full, and firm; skin dry; palpebræ nearly closed; *left* pupil dilated to nearly double the size of the right; pays no attention to surrounding objects, and could only be aroused with great difficulty. Was bled to the amount of ℥xxiv, and yesterday's treatment continued.

17th. Much worse; pulse very frequent, small and irritable; pupils dilated and insensible; skin clammy and inelastic; comatose.

He continued to grow worse, and died at two o'clock, P. M.

No autopsy could be obtained.

Case 2d. The following case I have extracted from my note book. It occurred in the New York Hospital during the term I was resident surgeon in that institution:

*Necrosis of Rib, Pleuropneumonia, Pericarditis.*—John McLareu, æt. 42, seaman, born in Ireland, admitted February 14th, 1840, complaining of a disease on the left side of his chest, attended with considerable pain, which had existed several months, being of late sufficiently severe to prevent him from following his usual occupation. On examination a flattened tumor, scarcely elevated enough to be perceptible at the first glance, was detected on the left and lower portion of the chest, just beyond the angle of the ribs. Pressure over this indicated a small elastic tumor, about an inch in diameter, flattened and attached apparently to the sixth rib. One or two smaller tumors, of a similar character, could be felt posterior to this, attached to the same rib. The bulging of the integuments, caused by these deep swellings, produced the flattened tumor on the surface, which was about three inches square. Pressure over this part gave the patient no uneasiness, and he was free from all evidence of disease in the thoracic viscera. He could assign no specific cause for the disorder, but thought he might have had a fall against that portion of his side.

About the 1st of March, a puncture was made with a lancet, through the swelling, down upon the principal of the elastic tumors above noticed. Some hemorrhage followed the incision, and a probe afterwards introduced reached the periosteum of the rib, which appeared to be hard and cartilaginous. No exposed bone could be detected, but a small quantity of thick purulent matter was pressed through the lips of the wound.

A day or two afterwards, the patient feeling himself able to work, went out of the hospital, but suppuration becoming abundant in the wound, he returned in eight or ten days.

On examination the matter was found to proceed from a deep and tortuous sinus, running in the direction of the rib, but external to it. No diseased bone has been as yet detected. The sinus was freely opened and dressed from the bottom, and was afterwards occasionally injected with a solution of sulphate of copper. Under this treatment the suppuration diminished, and the external incision contracted to a small fistulous opening.

April 22d. On examination with a probe, several pieces of detached bone were detected at the bottom of the fistula; the integuments were again divided, and several small pieces of loose bone were removed by the finger from the bottom of the wound. For two or three days subsequently small pieces of bone were detected, and removed at each dressing. The wound afterwards assumed a healthy character, and the patient's general health was to all appearances sound.

May 7th. The patient was, in the middle of the night, seized with a rigor, and on the following morning he was found laboring under considerable excitement ; pulse frequent, quick, and full ; skin hot and dry ; tongue coated with a white fur, and a severe pain in the head.

℞ Spts. Minder. ℥ viij.

Ant. Tart. gr. ij.

A tablespoonful to be given every two hours.

In the afternoon, his symptoms being more urgent, he was bled to the extent of ℥ xij, and the spts. Minder. and antimony continued.

9th. This morning the fever had abated somewhat, but as he complained of pain in his left side, he was freely cupped over that region. In the afternoon he appeared to be rather more comfortable.

10th. This morning he complained of severe pain in different parts of his body, but more particularly in his left side, and over the scorbiculus cordis ; the whole of the left side in a state of erysipelatous inflammation.—Ung. hyd. was applied to the inflamed surface, and the spts. Minder. and ant. tart. continued.

11th. This morning he appeared to be rather better, and continued so throughout the day.

In the afternoon, the spts. Minder. was discontinued, and he was directed to have a tablespoonful of the following mixture every two hours:

℞ Sulph. Magnes. ℥j.

Ant. Tart. gr. ij.

Aqua, ℥viij.

Misce.

12th. This morning he appeared to be worse, having passed a very restless night. The salts had not yet operated. His pulse was frequent and small, and the pain in the side very severe. Ordered cups to be applied sup: reg: dolor: and a purgative to be administered.

In the afternoon an enema was administered, which soon passed away, mixed with a small quantity of feces.

13th. Appears to be better; pulse rather fuller and less frequent; skin moist; very little pain, and erysipelas subsiding.

Solution of salts and antimony discontinued.

In the afternoon prot. chlor. hydrarg. gr. xx was given him.

14th. Passed another restless night, and complained this morning of a renewal of the pain.

In the afternoon another injection was given him, as the calomel had not operated, but this soon passed away, unmixed with any feculant matter. In the afternoon, ¼ gr. sulph. morph. was given him.

15th. Much about the same as yesterday. Spts. Minder. directed again.

In the afternoon it was first discovered that his pulse intermitted, and it evidently appeared that he was sinking. His voice was sepulchral; respiration short and labored; surface purplish, cool, moist, with clammy perspiration.

In the evening, sinapisms were applied to the calves of his legs, and brandy given him throughout the night.

16th. Much better than yesterday; skin hot and dry; pulse full, but intermitting.

In the afternoon, he appeared to be worse; surface again cool and purplish, with clammy sweat; pulse feeble and irregular. Had a simple injection, with tinct. aloes comp. ℥vj. added to it, given him, which passed away pure in a much less time than it took to give it. Brandy was again given him throughout the night.

17th. The erysipelas had entirely disappeared, but the posterior part of the body was very much congested.—Other symptoms same as before. Brandy discontinued. Had a blister applied to his left side, and tinct. digital. gtt. v given him every two hours.

He gradually got weaker throughout the day, and died at five o'clock, P. M.

*Autopsy, nineteen hours after death.*—On opening the chest the pericardium was found distended with serum, and the inner lining coated in all parts with a layer of plastic lymph, about a quarter of an inch in thickness, extending as well over the heart as within the pericardium proper; the heart itself in other respects was natural. The left lung had contracted a firm adhesion to the point of the chest opposite the diseased rib; the rest of the surface of the pleura was free, and coated like the pericardium, with a recent deposit of plastic lymph, that adhered but slightly to the part. The cavity of the pleura contained about half a pint of serum. The whole of the lower lobe of the left lung had been inflamed, and was in a state of red hepatization; the upper lobe was to all appearances healthy. The other viscera of the chest and abdomen presented no evidence of disease. On examining the rib it was found perforated by a hole large enough to contain the end of the finger, and to be deficient at its upper edge; this perforation, however, was prevented from communicating with the chest by the thickened periosteum, and by the internal adhesion already noticed. Another perforation was discovered to the sternal side of this, and extending about half way through the rib. The rib seems to have been fractured at these points a long time previous, and the callous appears to have been generated only at the lower portion, leaving the deficiencies that have given rise to these notches, which are now smooth and rounded off at the edges.

*Note.*—This patient had no cough during the whole of his disease, and his respiration was unembarrassed until within two days of his death. The disturbance in the circulation, the irregularity in the pulse, and a sudden sinking, with severe pain in the region of the heart, detected at that period, gave the first suspicions of pericarditis, which was detected after death.

